# Detailed Joint Region Analysis of the 7-Joint Ultrasound Score: Evaluation of an Arthritis Patient Cohort over One Year

**DOI:** 10.1155/2013/493848

**Published:** 2013-08-12

**Authors:** S. Ohrndorf, B. Halbauer, P. Martus, B. Reiche, T. M. Backhaus, G. R. Burmester, M. Backhaus

**Affiliations:** ^1^Department of Rheumatology and Clinical Immunology, Charité-Universitätsmedizin Berlin, 10117 Berlin, Germany; ^2^Department of Applied Biostatistics, UKT, University of Tübingen, 71070 Tübingen, Germany; ^3^Department of Medicine III, Nephrology and Rheumatology, Universitätsklinikum UKE, 20246 Hamburg, Germany

## Abstract

*Objective*. The main objective of this study was to evaluate the 7-joint ultrasound (US7) score by detailed joint region analysis of an arthritis patient cohort. *Methods*. The US7 score examines the clinically most affected wrist, MCP and PIP II, III, MTP II, and V joints for synovitis, tenosynovitis/paratenonitis, and erosions. Forty-five patients with rheumatoid arthritis (RA) (84.4%) and spondyloarthritis with polyarticular peripheral arthritis (PsA 13.3%; AS 2.2%) with a median disease duration of 6.5 yrs (range 7.5 mths–47.6 yrs) were included and examined at baseline and 3, 6, and 12 months after starting or changing therapy (DMARD/biologic). In this study, detailed US7 score joint region analysis was firstly performed. *Results*. The joint region analysis performed at baseline disclosed synovitis in 95.6% of affected wrists in the dorsal aspect by greyscale (GS) US where Grade 2 (moderate) was most often (48.9%) detected. Palmar wrist regions presented Grade 1 (minor) capsule elevation in 40% and Grade 2 (moderate synovitis) in 37.8%. Tenosynovitis of the extensor carpi ulnaris (ECU) tendon was found in 40%, with PD activity in 6.6%. Most of the erosions in MCP II were detected in the radial (68.9%), followed by the dorsal (48.9%) and palmar (44.4%) aspects. In MTP V, erosions were seen in 75.6% from lateral. *Conclusions*. Synovitis in GSUS was more often detected in the wrist in the dorsal than in the palmar aspect. ECU tendon involvement was frequent. Most erosions were found in the lateral scan of MTP V and the medial (radial) scan of MCP II.

## 1. Introduction

Objective imaging modalities are needed to detect the inflammatory and destructive processes in arthritic diseases such as rheumatoid arthritis (RA) and seronegative spondyloarthritis (e.g., psoriatic arthritis). In recent years, there have been numerous studies reporting early detection of soft tissue and bone processes in arthritic diseases and a high level of sensitivity in musculoskeletal ultrasonography (US) [1–8]. This imaging method allows disease activity and therapeutic response to be detected objectively and for immunosuppressive therapy to be adapted accordingly. As a result, better rheumatic disease outcomes might be achieved and structural damage prevented at earlier stages [9–13]. Due to rapid technical improvements, US has become the “extended diagnostic finger” in the rheumatologist's daily practice with high patient acceptability. Therefore, accurate assessment of joint inflammation such as synovitis and bone processes is extremely important and standardization is, therefore, essential. Recently, a novel 7-joint US (US7) score for use in daily rheumatologic practice has been developed which includes examination of the clinically most affected wrist, MCP II, III, PIP II, III, MTP II, and V, that is, the joints that are most frequently involved in RA [14, 15]. They are assessed for synovitis, tenosynovitis/paratenonitis, and erosions according to the EULAR criteria [16] and the OMERACT definition [17] including greyscale (GS) and power Doppler (PD) US. Synovitis and synovial/tenosynovial vascularity are scored semiquantitatively (grade 0–3) by PDUS according to Szkudlarek et al. [18]. Synovitis (effusion and synovial hypertrophy combined) in GSUS is analyzed semiquantitatively as described by Scheel et al. [19].

Tenosynovitis/paratenonitis as well as erosions in GSUS are registered as being absent (0) or present (1). The first publication describes the implementation of the US7 score in a nationwide project in order to prove its value and feasibility in daily rheumatologic practice. One hundred and twenty patients with RA (91%) and PsA (9%) were evaluated at three visits (baseline and after 3 and 6 months) using the US7 score. Clinical data (DAS28) and laboratory parameters (CRP and ESR) were also evaluated. All parameters were significantly reduced after three (except for the PDUS synovitis and erosions score) and six (except for the erosions score) months of therapy or change in immunosuppressive therapy (DMARDs and/or TNF*α* inhibitors versus DMARDs alone). The study demonstrated that the novel US7 score is not only a feasible score for monitoring disease activity in daily rheumatologic practice but also represents therapeutic response and is therefore sensitive to change [14]. This could also be presented by another US7 score validation study of a larger cohort of *n* = 432 RA patients [15]. 

The aim of the present study was to further validate this sum scoring system by detailed joint region analysis. To this end, one-year data of a monocenter subgroup of an arthritis patient cohort were analyzed. Primarily, the occurrence of pathologic findings such as synovitis, tenosynovitis/paratenonitis, and erosions in each joint region included in the US7 score was analyzed over the period of one year under certain therapies. Secondarily, each feature was evaluated for its predictive value for later erosions. Besides, US7 score data were compared to clinical and laboratory parameters.

## 2. Patients and Methods

Forty-five patients (77.8% female) suffering from RA (84.4%) and seronegative spondyloarthritis such as psoriatic arthritis (PsA; 13.3%) and ankylosing spondylitis (AS) with peripheral joint involvement (2.2%) with a median age (in years) of 56.0 (range 22–75 {95% CI: 45.5–65.0}) and median year of disease duration of 6.5 (range 7.5 mths–47.6 yrs {95% CI: 4.0–10.5}) were recruited from the Rheumatologic Outpatient Department of the Charité-Universitätsmedizin Berlin, Germany, from February 2007 to March 2009. The patient cohort consists of a monocenter subgroup from a large nationwide study. This study was approved by the ethical committee of Tuebingen, Germany (no. 199/2007BO2), and all included patients gave their informed consent. Required for inclusion was the indication for start or change of therapy to DMARDs and/or biologics (because of either disease activity or medical side effects), as well as a minimum age of 18 years and a statement of agreement. 57.8% of the included patients were positive for rheumatoid factor IgM (RF IgM) and 55.6% for anticitrullinated antibodies (ACPA). Clinical, laboratory, and US data were evaluated before (baseline) starting or changing therapy (DMARD and/or biologica) and after 3, 6, and 12 months. Following the baseline examination, 57.8% of the patients received DMARD and TNF*α* inhibitor combination therapy, 26.7% DMARDs only, and 15.5% TNF*α* inhibitor monotherapy.

### 2.1. Clinical and Laboratory Assessment

 For current disease activity evaluation, the disease activity score 28 (DAS28) was assessed at each patient's visit. Furthermore, erythrocyte sedimentation rate (ESR; normal level < 20 mm/h) and C-reactive protein (CRP; normal level < 5 mg/L) levels were measured at baseline and after 3, 6, and 12 months. 

### 2.2. Imaging Assessment

**Figure 1:**
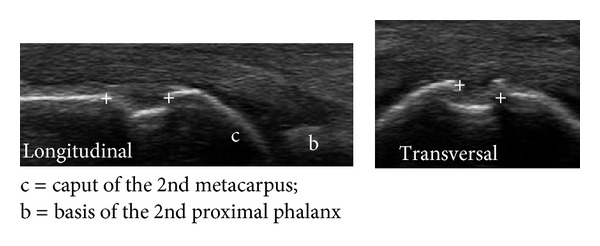
Erosion in the 2nd MCP joint from dorsal.

**Figure 2:**
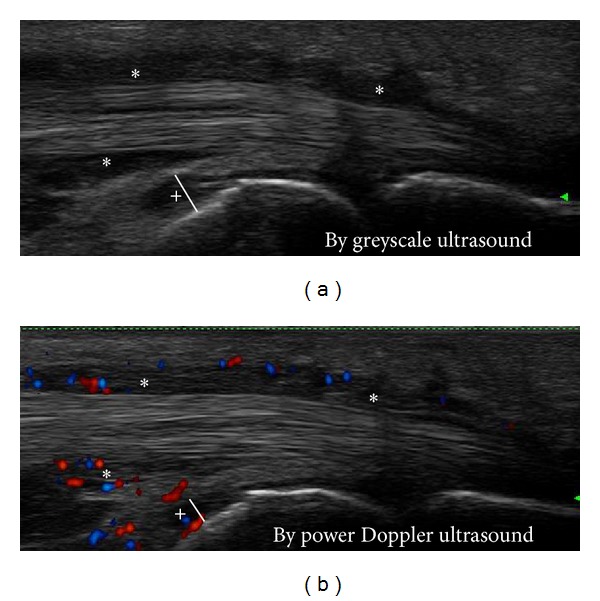
Tenosynovitis (∗) of the 2nd flexor tendon and synovitis (+) of the 2nd MCP joint—longitudinal scan.

For the *ultrasound* examination, the novel US7 score [14, 15] was applied at each visit. This score includes US evaluation of the following joints of the clinically most affected hand and forefoot: wrist, MCP II, III, PIP II, III, MTP II, and V which are assessed for synovitis, tenosynovitis/paratenonitis, and erosions. Synovitis and synovial/tenosynovial vascularity are scored semiquantitatively (grade 0–3) by GSUS and PDUS and tenosynovitis as well as erosions for their presence (0/1). In this study, the *wrist* was examined in the dorsomedian, ulnar, and palmar aspects for synovitis and tenosynovitis in GSUS and PDUS and for erosions. The finger joints *MCP II *and* III* were assessed in the dorsal aspect for synovitis in PDUS, for paratenonitis in GSUS and PDUS, also for erosions (see an example of an erosion in the 2nd MCP joint in Figure 1), then in the medial (radial) aspect for erosions (only MCP II), and in the palmar aspect for synovitis and tenosynovitis in GSUS and PDUS (an example of tenosynovitis and synovitis in the 2nd MCP joint detected by GSUS and PDUS is given in Figure 2) as well as for erosions. *PIP joints II *and* III* were examined in the dorsal aspect for synovitis in PDUS and for erosions and in the palmar aspect for synovitis in GSUS and PDUS as well as for erosions. The toe joints, *MTP II and V*, were examined in the dorsal aspect for synovitis in GSUS and PDUS and for erosions and in the plantar and lateral (only MTP V) aspects for erosions. Sum scores for synovitis, tenosynovitis/paratenonitis, and erosions were composed for each time of US assessment. The scoring range for the GS synovitis score was 0–27, for the PD synovitis score 0–39, for the GS tenosynovitis score 0–7, for the PD tenosynovitis score 0–21, for the erosions score 0–14 excluding wrist examination, and 0–17 including wrist examination. Sum scores excluding the forefoot joints examination (“US5” score) were also performed in order to determine whether the scoring system, without the forefoot joints MTP II and V, is sensitive to change. 

Furthermore, a detailed joint region analysis was performed at baseline and after 3, 6, and 12 months calculating the amount of pathologic findings in each joint region included. Two expert sonographers (MB, SO) performed the US7 score examination. The ultrasonographers were both aware of the treatment and the treatment decision. There was no blinding to treatment in this study.

At baseline and after 12 months, conventional *radiographic scans* of both hands and forefeet in two planes were performed according to German recommendations. The presence of erosions was qualitatively assessed (0/1) by the Steinbrocker score (Steinbrocker score ≤ 1 = 0, Steinbrocker > 1 = 1) [20]. 

### 2.3. Statistical Analysis

The statistical calculation was carried out using the statistical software program SPSS 18.0 (SPSS, Chicago, Illinois, USA). Median values and interquartile ranges as well as the amount of pathologic findings (%) were calculated, and changes were subjected to the 2-sided exact Wilcoxon test. The longitudinal differences of laboratory, clinical, and US parameters were correlated using Spearman nonparametric correlation coefficients. For the calculation of predictive values, linear regression analysis was performed, and predictors for dependent variables (US7 erosion score) were calculated. For the calculation of the prediction of later erosions by conventional radiography, logistic regression was used; fit was assessed by the Hosmer-Lemeshow test. Variable selection was applied with inclusion probability assessed by the Rao Score test but significance within the model by the Wald test. Statistical significance (*P*) was set at the *α* ≤ 0.05 level. No adjustment for multiple testing was applied. 

## 3. Results

### 3.1. Detailed Joint Region Analysis

Baseline joint region analysis disclosed synovitis in 95.6% affected *wrists* in the dorsomedian aspect by GSUS, where Grade 2 (moderate) was detected most often (48.9%). Power Doppler activity was found in 64.4% with Grade 2 (35.6%) being detected most often. Furthermore, erosions in this joint region were seen in 68.9% of the patients. Tenosynovitis in GS mode was detected in 17.8% in this joint region with PD activity in 4.4%. Palmomedian wrist regions presented Grade 1 (minor) capsule elevation in 40%, and Grade 2 (moderate synovitis) in 37.8% with PD activity of 11.1%. Erosions in this region were detected in 57.8% of the cases. Tenosynovitis in this joint region was seen in only 6.7% without any PD activity. Tenosynovitis of the extensor carpi ulnaris (ECU) tendon detected by GSUS was found in at least 40% of the joints examined, with PD activity of 6.6%. Erosive changes were seen in 44.4% of the ulnar wrist regions.

Regarding *finger joint* examination, synovitis was determined in 95.6% both in MCP joint II and III in the palmar aspect by GSUS, whereby Grade 2 was detected most often in MCP II (33.3%) and Grade 1 (51.1%) in MCP III. In both joints, PD activity was more frequently observed in the dorsal aspect than in the palmar aspect (MCP II: 24.4% versus 17.8%; MCP III: 24.4% versus 4.4%). Most erosions in MCP II were detected in the medial (radial) aspect (68.9%), followed by the dorsal aspect (48.9%) and the palmar aspect (44.4%). In MCP III, erosions were seen in 44.4% of the joints in the dorsal aspect and in 35.6% in the palmar aspect. Tenosynovitis was detected in 15.6% in MCP II and in 6.7% in MCP III, and paratenonitis was (only) found in 4.4% in MCP II and in 2.2% in MCP III. In the PIP joints included, the following observations were made: PIP II synovitis by GSUS was detected in 81.8% of the joints in the palmar aspect where Grade 1 with 43.2% was detected most often. PIP III was affected by GS synovitis in 68.9% of the cases with Grades 1 and 2 reaching the same incidence (24.4%). In both joints, PD activity was more frequently seen in the dorsal than in the palmar aspect (PIP II: 14.3% versus 13.6%; PIP III 13.3% versus 11.4%). Furthermore, erosions were detected more often in the dorsal aspect than in the palmar aspect (PIP II 51.1% versus 42.2%; PIP III 46.7% versus 31.1%). 

**Figure 3:**
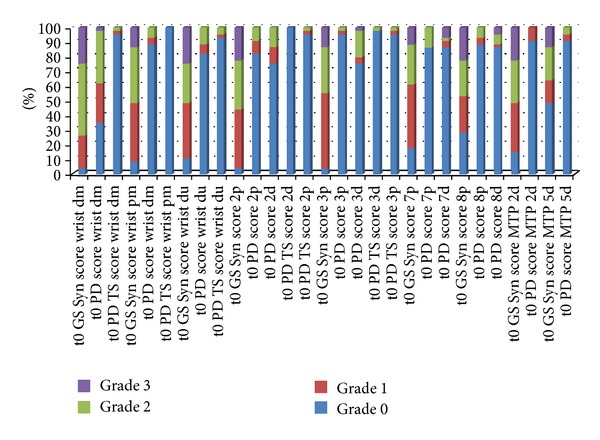
Semiquantitative analysis of each joint region included in the US7 score (without erosions)—baseline (t0). *Legend: *Syn = synovitis; TS = tenosynovitis/paratenonitis; GS = greyscale; PD = power Doppler; dm = dorsomedian; pm = palmomedian; du = dorsoulnar; 2p = MCP II palmar; 2d = MCP II dorsal; 3p = MCP II palmar; 3d = MCP III dorsal; 7p = PIP II palmar; 7d = PIP II dorsal; 8p = PIP III palmar; 8d = PIP III dorsal.

**Figure 4:**
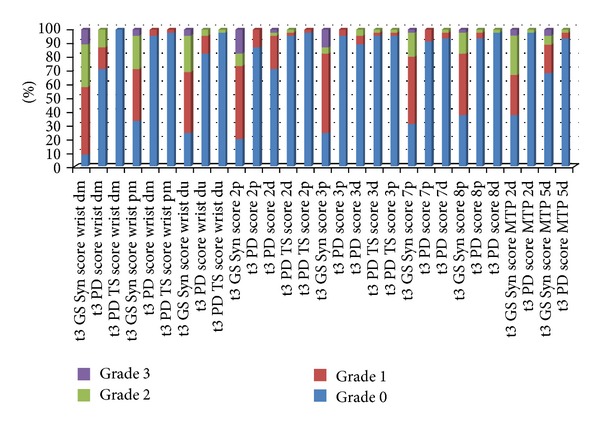
Semiquantitative analysis of each joint region included in the US7 score (without erosions)—one-year followup (t3). *Legend: *Syn = synovitis; TS = tenosynovitis/paratenonitis; GS = greyscale; PD = power Doppler; dm = dorsomedian; pm = palmomedian; du = dorsoulnar; 2p = MCP II palmar; 2d = MCP II dorsal; 3p = MCP II palmar; 3d = MCP III dorsal; 7p = PIP II palmar; 7d = PIP II dorsal; 8p = PIP III palmar; 8d = PIP III dorsal.

In the *toe joints*, MTP II was affected by GS synovitis in 84.4% of the cases with PD activity in 33.3% (dorsal examination). Erosions in this joint were more often detected in the dorsal aspect (46.7%) than in the palmar (22.2%). In MTP V, synovitis was seen in 51.1% of the joints with PD activity in only 8.9% (dorsal examination). Erosions in MTP V were most frequently detected in the lateral aspect (75.6%), followed by the dorsal (57.8%) and palmar aspects (44.4%). See Figure 3 for the semiquantitative analysis of each joint region included in the US7 score (baseline). The one-year joint region analysis is presented in Figure 4.

The following joint regions were the ones which were most significantly changing under new treatment regime over one year: PD positive synovitis of the dorsomedian wrist and GSUS synovitis in the palmar MCP III joint region and in the palmar PIP III region (each *P* < 0.001).

### 3.2. Prediction of Erosions after One Year Based on the US7 Score and Laboratory and Clinical Parameters

For the prediction of erosions in both hands and forefeet *detected by conventional radiography* after one year, certain predictors were approved. Considering the US7 score features, the synovitis score by GSUS at baseline was a significant predictor (*P* < 0.05) for erosions on radiographs after one year. Furthermore, initial erosions in the US7 score (excluding and including wrist examination) were highly significant predictors for erosions in conventional radiography after one year (*P* < 0.001). In multivariate analysis, baseline US5 erosions sum score (*P* < 0.001, odds ratio = 2.31 {95% CI: 1.46–3.65}) and US7 GS tenosynovitis/paratenonitis sum score (Rao score test *P* = 0.021, Wald test *P* = 0.056, odds ratio = 2.45, {95% CI: 0.98–6.15}), which was not significant in univariate analysis, were included by the variable selection procedure. The Hosmer-Lemeshow test revealed a good fit of the final model (*P* = 0.32). Other US7 score features such as the synovitis score in PDUS and tenosynovitis/paratenonitis score did not significantly predict erosions by radiographs after one year. Laboratory (CRP, ESR) and the clinical parameter DAS28 were not significant predictors of erosions in conventional radiography either.

For the prediction of erosions *detected by the US7 score* (erosion score including the wrist) after one year, the following significant baseline predictors were evaluated: DAS28 (*P* < 0.05), US7 synovitis score in GSUS (*P* = 0.001), and PDUS (*P* < 0.05), of which the synovitis score in GSUS was the only multivariately significant predictor. Laboratory data (CRP, ESR) and antibodies (RF IgM and ACPA positivity) as well as tenosynovitis/paratenonitis scores by GSUS and PDUS did not significantly predict erosions in ultrasonography. The fit of this linear model was satisfactory; quadrat terms were not significant and residuals approximately normally distributed.

For the prediction of the erosions score excluding wrist examination, similar results were obtained as follows: US7 synovitis score in GSUS (*P* = 0.002) and PDUS (*P* = 0.001). The DAS28 was not predictor for significant erosions if excluding the wrist examination for erosions (*P* = 0.102). 

### 3.3. Laboratory, Clinical, and US7 Score Data over One Year

Laboratory (ESR, CRP), clinical (DAS28), and US7 (also without forefoot joints examination—“US5”) score data were evaluated at each assessment time, and changes to baseline were examined (Tables 1 and 2). 

The laboratory parameters, ESR and CRP, significantly decreased 3 months after starting or changing therapy. After 6 and 12 months, median ESR and CRP did not change significantly versus baseline examination. Median DAS28 significantly decreased from 4.8 at baseline to 4.1 (after 3 months), then from 3.7 (after 6 months) to 3.8 (after 12 months; *P* < 0.05 in each case to baseline). A significant reduction of the median synovitis score in GSUS was also observed at each assessment time from 13.0 initially to 9.0 (30.8%; *P* < 0.05) 12 months later. The median synovitis score in PDUS decreased significantly from 2.0 initially to 1.0 after 6 months (50%; *P* < 0.05). The median tenosynovitis/paratenonitis score in GSUS (initially 1.0) was significantly reduced after 6 and 12 months (each 0.0; *P* < 0.05) of US examination. The median tenosynovitis/paratenonitis score in PDUS remained the same over the period of one year. According to the erosions score, both excluding and including wrist examination, a significant reduction was observed from 7.0 initially (excluding the wrist) and 8.0 (including the wrist) to 5.0 and 6.0 after 12 months, respectively (*P* < 0.05 in each case; US7 score data, Table 1). Excluding the forefoot (MTP II and V) US examination, the same statistical analysis compared to the US7 score analysis was performed (“US5” score data, Table 2). The GSUS synovitis score significantly decreased at each assessment time from 11.0 initially to 7.0 (36.4%, *P* < 0.05) after 12 months. A significant reduction of the median synovitis score in PDUS was first seen after 6 months from 2.0 to 1.0 (50.0%; *P* < 0.05), then up to 1.7, but still significantly reduced to baseline (15.0%; *P* < 0.05). Furthermore, the erosions score for the hand, both excluding and including wrist examination, was significantly reduced from 4.0 initially (excluding the wrist) and 6.0 (including the wrist) to 2.9 and 5.0, respectively (*P* < 0.05 in each case).

At baseline, 49% of the patients had erosions in both hands and 35% in both feet in conventional radiographs. After 12 months, 51.1% of the patients had erosions in both hands and 26.7% in both feet.

### 3.4. Correlations between the US7 Score and Laboratory and Clinical Parameters over 12-Month Followup

There was a significant correlation between changes in the US7 score obtained by GSUS and the ESR through 12 months of followup (GSUS7/ESR: *r* = 0.31; *P* < 0.05). No other significant correlations were detected between the US7 score obtained by GSUS and PDUS compared to laboratory and clinical parameters. No significant positive correlation coefficients were found between the “US5” score (excluding the forefoot joints) data and clinical and laboratory parameters.

## 4. Discussion

The aim of the present study was to further validate the US7 score by a detailed joint region analysis. For this purpose, the incidence of pathologic findings such as synovitis, tenosynovitis/paratenonitis, and erosions in each included joint region were evaluated and analyzed over the period of one year. Secondarily, different components of the US7 sum score were examined for their ability to predict later erosions. Furthermore, one-year data of the US7 score evaluated in this subgroup of patients were compared to clinical (DAS28) and laboratory (CRP, ESR) parameters. 

Regarding the detailed joint region analysis, the most affected joints/joint regions affected by synovitis (detected by GSUS) were the wrist in the dorsal aspect and the MCP II in the palmar aspect, whereby the wrist was more severely affected. Furthermore, power Doppler activity > Grade 1 was mostly seen in the dorsal wrist joint. It was also one of the joint regions, which was most significantly changing under new treatment regime. Recently, Ellegaard et al. presented a study in which they proposed that a standardized color Doppler US examination of the wrist joint as the only target joint was very helpful in the detection of disease activity with a high correlation to CRP, ESR, swollen joint count, and DAS28 [21]. 

For the US7 score evaluation, synovitis in grey scale was only evaluated in the palmar aspect for the finger joint examination. In view of the findings of the study by Scheel et al. [19] in which synovitis was most often presented in the palmar proximal side of the finger joints (86%), we decided to exclude the dorsal compartment examination by GSUS, especially in the case of time period. This phenomenon was recently determined in a study by Vlad et al. in which the palmar finger joint regions (MCP and PIP II-V) showed better correlation to clinical evaluation (CDAI and SDAI) than the dorsal examination [22]. Furthermore, Dougados et al. were not able to demonstrate that musculoskeletal US was more sensitive than clinical examination [23]. This may be due to the fact that synovitis was only examined in the dorsal aspect in this study; a region which can easily be evaluated by clinical examination. However, for the detection of synovitis by PDUS, the dorsal side of the finger joints should be included, as PD activity was found there more often.

Most erosions were found in the ulnar scan of the MTP V and the radial scan of the MCP II joint. This distribution of erosions was shown by a French US working group before presenting new semiquantitative erosions score with good correlation to radiography. This group also found erosions primarily in MTP V, followed by MCP II [24].

The predictive value of the US7 erosions score after one year was most significant for the synovitis score in GSUS and less, but still significant, for the synovitis score in PDUS and the DAS28 clinical score (if detecting erosions in the wrist). The predictive value for radiographic erosions after one year was most significant for the US7 erosions score at baseline indicating that the initial US erosions score did have the highest predictive power for the disease outcome. Furthermore, the synovitis score in GSUS did significantly predict later erosions on radiography. Therefore, permanent reduction of synovitis needs to be the most important aim of the therapeutic concept in order to prevent the destructive process of the disease. In the present study, tenosynovitis, especially ECU tenosynovitis, was not predictive for later erosions. However, this could recently be presented by Lillegraven et al. [25] who found ECU tenosynovitis to be the only independent predictive value for later erosions in the hands in MRI. The present calculation probably results from the fact that these arthritis patients already had a long disease history at study onset, and the erosions were only detected by conventional radiography. It is a known fact that MRI is more sensitive in the detection of erosions than radiography.

One-year data of the US7 score compared to clinical (DAS28) and laboratory (CRP, ESR) parameters disclosed a significant reduction in the one-year data in most of its components; that is, this novel sum score is sensitive to change under certain therapies. Even *without* examination of the forefoot joints MTP II and V (“US5”score), this scoring system still worked well as the same statistically significant changes were calculated between followup and baseline. Consequently, additional time was saved by omitting the forefoot US examination which would make this score even more feasible. However, correlation analysis of changes to baseline only presented significant coefficients between the synovitis score in GSUS and ESR when the forefoot examination (US7 score) was included but not without it (“US5” score). Therefore, inclusion of the forefoot (MTP II and V) examination may contribute to higher sensitivity of this composite scoring system. Furthermore, for the individual patient it might be very important to include these joints even so the average statistical comparison of populations and/or time points suggest to shorten the examination accordingly.

Regarding the US7 erosion score, a statistically significant reduction over the examination period of one year was detected. Therefore, a potential “healing effect” of bone lesions beyond immunosuppressive therapy needs to be discussed. This phenomenon was already described by Rau et al. for radiographic erosions [26, 27] and was recently evaluated by Finzel et al. for erosions detected by microcomputed tomography (*μ*CT) [28]. In the Finzel et al. paper, bone erosions in RA patients receiving either tumor necrosis factor inhibitors or methotrexate were assessed by micro-CT imaging. After one year, patients taking TNF*α* inhibitors showed partial recovery in terms of a decrease in the mean depth of erosions while the mean width remained the same. In contrast, patients taking only methotrexate demonstrated an increase in the main depth and width of the erosions. To our knowledge, a reduction of erosions has not yet been described for musculoskeletal ultrasonography. One reason for the reduction of erosions might be due to the fact that, with less synovitis in the follow-up examinations, the delay path to bone surface is reduced so that the erosions are no longer readily detectable. Therefore, the erosions would only seem to be reduced but no longer be reproduced. However, interobserver reliability for the US7 erosions score was *κ* = 0.45 [29], at least, which means that there is moderate agreement concerning the detection of this pathology. Furthermore, in a recent study by Døhn et al. it was shown that erosions evaluated by US are true erosions compared to computer tomography [30]. Most of the patients participating in the present study also received TNF*α* inhibitor therapy (57.8% in combination with DMARDs and 15.5% alone), a fact that makes the results even more plausible. Therefore, the healing effect of erosions is also detectable by musculoskeletal US, a new aspect for this imaging modality. 

Summarizing the findings, it could be said that both the US7 and the “US5” scores are feasible sum scoring systems for use in daily rheumatologic practice. But because paratenonitis was a rare finding, it might not be a necessary component in the US7 scoring system and could therefore be excluded.

Further studies, especially with a homogenous group of early RA/arthritis patients, need to follow in order to examine the role of the additive value of the US7 score compared to conventional clinical and serological parameters, especially with regard to the outcome parameters (e.g., its value as a predictor of later erosions or to characterize patients who do not respond to certain therapies (i.e., TNF*α* inhibitors), etc.). Besides, the question concerning the meaning of subclinical disease activity detected by musculoskeletal US should be further discussed, for example, in case of therapeutic escalation. Furthermore, thresholds for the different components of the US7 score need to be analyzed and defined in order to standardize this composite scoring system more thoroughly.

Regarding the observed positive overall effect comparing followup with baseline, we have to point out that this was not a controlled therapeutic study. Especially we cannot exclude that patients entered the observation at a peek of disease burden, and in the followup, a regression to the mean was observed. Furthermore, the factor of a “mix” of different longstanding arthritis patients, though mainly RA patients, is the main limitation of the study.

## Figures and Tables

**Table 1:** Laboratory, clinical, and US7 score data over one year.

*n* = 45 patients	Baseline	After 3 months	After 6 months	After 12 months
ESR (mm/h)	24.5 (5–82) {95% CI: 14.3–36.0}	14.0 (3–74)* {95% CI: 8.0–29.5}	19.0 (3–120) {95% CI: 7.5–38.3}	14.5 (3–94) {95% CI: 9.8–36.8}
CRP (mg/L)	4.1 (0.2–119) {95% CI: 2.0–9.2}	3.0 (0.3–34.8)* {95% CI: 1.1–5.9}	3.0 (0.2–38.1) {95% CI: 1.0–6.8}	3.6 (0.0–65) {95% CI: 1.4–7.5}
DAS28	4.8 (1.6–8.0) {95% CI: 4.1–6.1}	4.1 (1.4–7.6)* {95% CI: 2.9–4.9}	3.7 (1.1–6.8)* {95% CI: 2.5–5.2}	3.8 (1.3–7.1)* {95% CI: 2.8–4.7}
Synovitis score in GSUS (0–27)	13.0 (7–25) {95% CI: 0.5–6.5}	10.0 (3–22)* {95% CI: 6.5–15.0}	10.0 (2–18)* {95% CI: 7.0–13.5}	9.0 (0–20)* {95% CI: 6.0–12.0}
Synovitis score in PDUS (0–39)	2.0 (0–16) {95% CI: 0.5–6.5}	1.0 (0–20) {95% CI: 0.0–5.0}	1.0 (0–18)* {95% CI: 0.0–3.0}	2.0 (0–9)* {95% CI: 0.0–2.5}
Tenosynovitis/paratenonitis score in GSUS (0–7)	1.0 (0–5) {95% CI: 0.0–1.0}	0.0 (0–5) {95% CI: 0.0–1.0}	0.0 (0–3)* {95% CI: 0.0–1.0}	0.0 (0–5) {95% CI: 0.0–1.0}
Tenosynovitis/paratenonitis score in PDUS (0–21)	0.0 (0–8) {95% CI: 0.0–0.0}	0.0 (0–8) {95% CI: 0.0–0.0}	0.0 (0–5) {95% CI: 0.0–0.0}	0.0 (0–6) {95% CI: 0.0–0.0}
Erosions score excluding the wrist (0–14)	7.0 (0–14) {95% CI: 4.0–9.0}	6.0 (1–14) {95% CI: 4.0–9.0}	6.0 (0–14) {95% CI: 4.0–9.0}	5.0 (0–11)* {95% CI: 3.0–7.0}
Erosions score including the wrist (0–17)	8.0 (0–17) {95% CI: 5.0–11.0}	7.0 (2–17) {95% CI: 6.0–11.5}	8.0 (0–17) {95% CI: 5.0–11.5}	6.0 (1–14)* {95% CI: 5.0–9.0}

*P value < 0.05 to baseline examination by 2-sided exact Wilcoxon test.

**Table 2:** “US5” (without forefoot results) score data over one year.

*n* = 45 patients	Baseline	After 3 months	After 6 months	After 12 months
Synovitis score in GSUS (0–21)	11.0 (7–19) {95% CI: 8.0–13.0}	9.0 (2–18)* {95% CI: 6.0–12.5}	9.0 (1–16)* {95% CI: 5.5–10.5}	7.0 (0–20)* {95% CI: 5.0–10.0}
Synovitis score in PDUS (0–33)	2.0 (0–15) {95% CI: 0.5–6.0}	1.0 (0–17) {95% CI: 0.0–5.0}	1.0 (0–18)* {95% CI: 0.0–3.0}	1.7 (0–7)* {95% CI: 0.0–2.5}
Erosions score excluding the wrist (0–9)	4.0 (0–9) {95% CI: 2.0–6.0}	3.0 (0–15) {95% CI: 2.0–6.0}	4.0 (0–9) {95% CI: 2.0–6.0}	2.9 (0–7)* {95% CI: 1.0–4.5}
Erosions score including the wrist (0–12)	6.0 (0–12) {95% CI: 3.0–8.5}	5.0 (1–12) {95% CI: 4.0–9.0}	6.0 (0–12) {95% CI: 3.0–8.5}	5.0 (0–10)* {95% CI: 3.0–7.0}

*P value < 0.05 to baseline examination by 2-sided exact Wilcoxon test.

## Abbreviations

US7 score: 7-joint ultrasound score
“US5” score: 5-joint ultrasound score (without forefoot)
RA: Rheumatoid arthritis
PsA: Psoriatic arthritis
AS: Ankylosing spondylitis
DMARD: Disease modifying antirheumatic drugs
GSUS: Grayscale ultrasound
PDUS: Power Doppler ultrasound
MCP: Metacarpophalangeal
PIP: Proximal interphalangeal
MTP: Metatarsophalangeal
EULAR: European League Against Rheumatism
OMERACT: Outcome measure in rheumatology clinical trial
ESR: Erythrocyte sedimentation rate
CRP: C-reactive protein
DAS28: Disease activity score of 28 joints
CDAI: Clinical disease activity index
SDAI: Simplified disease activity index
CT: Computer tomography
MRI: Magnetic resonance imaging.
